# “I live by myself, but at least God is with me”: The effects of faith-based identity on mental health during transitions in social and legal status to the United States among Korean/Korean American immigrants

**DOI:** 10.1186/s12889-025-25268-6

**Published:** 2025-11-26

**Authors:** Chulwoo Park, Airi Irene Trisnadi, Patricia Angelica

**Affiliations:** 1 Department of Sociology, Anthropology, and Public Health, University of Maryland, Baltimore County, Baltimore, MD 21250 USA; 2 Department of Psychology, San José State University, San Jose, CA 95192 USA; 3 Department of Public Health and Recreation, San José State University, San Jose, CA 95192 USA

**Keywords:** Faith-based identity, Christianity, Korean american immigrant, Online survey, In-depth interview, Mixed methods study

## Abstract

**Background:**

Among Korean/Korean American immigrants, the tenth-largest immigrant population in the United States, approximately 30% of them reside in California. Christianity has played a significant role in supporting their mental well-being; however, little is known about how faith-based identity influences their mental health dealing with legal status challenges while adjusting to life in the Bay Area. Thus, this study sought to understand how faith-based identity, specifically Christianity, supports Korean/Korean American immigrants in overcoming mental health challenges and enhancing a sense of belonging during transitions in their social and legal status in the United States.

**Methods:**

We used a mixed methods approach. First, we collected a quantitative online survey. Second, we invited a total of 24 survey participants to conduct a qualitative in-depth interview, the majority of whom participated in person (22 out of 24). Eligible participants met the following inclusion criteria: (1) both of their parents were of Korean ethnicity, regardless of nationality; (2) 18 years of age or older; (3) regular attendance at a Korean church in the San Francisco Bay Area; and (4) having an ability to read and understand English for both the survey and interview.

**Results:**

We extracted four themes. First, most participants had been exposed to Christianity before coming to the United States, but their initial faith was generally weak. Second, later on, they deepened their Christian identity through various religious practices, such as attending Christian retreats, reflecting on beliefs, and praying to God. Third, by receiving support from the church and living with Christian teachings, they overcame challenges through faith, which positively contributed to their mental well-being. Lastly, many participants expressed a sense of God’s calling in the San Francisco Bay Area, which provided them a clear direction for pursuing and achieving their goals.

**Conclusions:**

The study explains how Christianity influences the psychological well-being of Korean/Korean American immigrants in the United States. It highlights the importance of further understanding about the role of faith-based identity among immigrant populations, in order to support their mental health when experiencing transitions in their social and legal status in the United States.

## Introduction

Korean/Korean American immigrants from South Korea (hereafter referred to as Korean immigrants) constitute the tenth-largest immigrant population and the fifth largest Asian group in the United States [1, 2]. According to the 2023 American Community Survey collected by the U.S. Census Bureau, the total population of Koreans alone or in any combination is 2,023,517 [3]. It is estimated that 30% of the Korean immigrant population resides in California, the highest percentage among all states, followed by New York (8%) and New Jersey (7%) [2]. Silicon Valley is among the top 10 metropolitan areas with significant Korean immigrant populations [2]. Immigrants undergo an adjustment period as they encounter unfamiliar factors such as weather, landscape, language, values, and beliefs [4]. These challenges can lead to significant stress, homesickness, and feelings of isolation [5], thereby increasing the risk of developing mental health issues. Major depression is a common mental illness in the United States and is prevalent among immigrant populations [5, 6]. The California Public Policy Institute found that immigrants who were more proficient in English and had permanent legal status reported lower psychological distress [5].

Despite the challenges they face, Asian immigrants, including Korean immigrants, are generally resistant to receiving mental health services [7]. However, churches, particularly ethnic Korean churches, have positive influences on Korean immigrants’ mental health. Korean ethnic churches contribute to Korean immigrants’ mental health by reinforcing traditional customs and values and serving as crucial support systems in the new environment. Korean immigrants rely on these churches for support, including spiritual counseling, as many believe that church pastors are trusted figures with whom they can openly communicate [8].

According to Pargament’s theory of religious coping (1997), there are several attributes of religious and spiritual belief on coping strategies, which are categorized as cognitive, behavioral, and interpersonal [9]. Pargament outlines five fundamental functions of religious coping: making meaning of events, having control over challenges, finding comfort, connecting with peers, and navigating life transitions. In addition, Pargament emphasizes that religious beliefs and practices can be associated with both positive and negative coping mechanisms. Positive religious coping includes seeking spiritual support and strengthening spiritual connection, which contributes to problem-solving and overcoming life difficulties. In contrast, negative religious coping may involve the lack of support from religious communities which lead to interpreting life experiences as consequences or punishments from God. Members of the church community provide mental and emotional support for immigrants as Korean church members often form strong relationships through their shared culture [10]. They provide new Korean immigrants with a sense of belonging and connection to their ethnic background, thus offering psychological and emotional support [10]. Additionally, they offer immigrants an environment for networking and social interactions with other Korean immigrant members [10].

Addressing the needs of the immigrant population has become a pressing issue for Korean churches. Over time, Korean churches have created a unique social and cultural impact, playing a crucial role by offering essential services to Korean immigrant communities. For example, these services may include airport pickups and assistance with obtaining driver’s licenses and social security cards. Church leaders and volunteers dedicate significant time and energy to create a welcoming environment for Korean immigrants. Many non-believing Korean immigrants then started to attend a Korean church in America and eventually converted to Christianity [11]. Christianity plays a central role in the religious lives of Korean immigrants, shaping how faith intersects with mental health. According to the Pew Research Center, nearly 6 among 10 Korean Americans (59%) identify as Christian, which include Protestants (46%) and Catholics (11%), with fewer identifying as Buddhist (3%) or as religiously unaffiliated (34%) [12, 13]. Christianity is reported to be significantly more prevalent among Korean Americans (59%) than among Asian Americans (34%) [14].

The San Francisco Bay Area (hereafter referred to as the Bay Area) remains the most unchurched and de-churched compared to any other metropolitan area in the country [15, 16]. Despite some understanding of immigrant experiences, little is known about how Christian identity—defined as a multidimensional construct involving religious belief, practices, and social connections and used interchangeably hereafter—affects the mental health of Korean immigrants dealing with social and legal status challenges while adjusting to life in the Bay Area. While previous research has primarily focused on measuring mental health outcomes among immigrants, this study takes a mixed methods approach to explore the experiences of Korean immigrants, focusing on the role of Christian faith during critical transitions in social and legal status that are often accompanied by stress and uncertainty. Thus, the purpose of this study was to investigate how faith-based identity affects mental health during transitions in social and legal status, before and after adjusting to the new environment in the United States, among Korean/Korean American immigrants. We adopted a multidimensional understanding of Christian identity that extends beyond personal belief, which encompasses religious practices such as worship services and prayers, social connection involving church membership, and identity [17, 18]. This approach reflects how Korean immigrants experience Christianity both as a faith system and as a social structure that provides community, cultural continuity, and support during immigration transitions. In this study, “religion” was framed not merely as belief in God but as a multidimensional aspect that encompasses religious practices (e.g., worship and service), social connection (e.g., church membership and peer relationship), and identity. This reflects the way in which Korean immigrants experience Christianity both as a faith system and as a social structure that provides community, cultural continuity, and support during transitions.

## Materials and methods

### Study design

We designed a sequential explanatory mixed methods research study consisting of two components: (1) a quantitative online survey, followed by (2) a qualitative in-depth interview. Using Qualtrics (Qualtrics International Inc., Provo, UT), the quantitative survey was administered first to collect the information of (a) Demographics, (b) Health, (c) Legal status, and (d) Religious behaviors, which took approximately 10–15 min to complete. We extracted questions from Wave VI of the Baylor Religion Survey (2021), Values and Beliefs of the American Public Survey for the categories of demographics, health, and religious behaviors [19], and then modified them as necessary and included additional questions applicable to our survey. For legal status questions, we asked the type of visa when participants first came to the United States, current visa status when they filled in the survey, as well as their intention to stay in the United States and whether belief in Christianity helped them overcome challenges when obtaining stable legal status using a 5-item Likert scale of strongly agree to strongly disagree.

Among those who completed the survey, a total of 24 individuals agreed to participate in the one-on-one in-depth qualitative interview. We used quantitative data from those who completed a subsequent in-depth interview. This approach ensures that the demographics, health, legal status, and religious behaviorsinformation presented reflect the same group of individuals who provided in-depth qualitative data. The interviews were designed to provide both deductive and inductive insights that were not obtained from the survey. From March to July 2023, the survey data were collected, and subsequently, one-on-one in-depth interviews were conducted. A rationale for this study was to include individuals who attend the church to better understand the role of Christianity and the Korean church community among Korean immigrants. We focused specifically on Korean immigrants in the Bay Area due to the region’s large Korean population and cultural diversity, as well as our proximity to the area, which made it convenient to meet participants in person and strengthened the qualitative data collection. Using a mixed methods research design, we incorporated both the quantitative data we collected from the online survey for descriptive analysis and the qualitative data from the interview portion of the study to identify depth and meaning to the participants’ narratives. The combination of quantitative and qualitative information offered greater insights and allowed better interpretations. While the quantitative data were limited to descriptive statistics due to the small sample size, the data provided contextual understanding of patterns within the participant group, such as the prevalence of certain experiences or beliefs. The qualitative interview offered detailed narratives of participants’ lived experiences which identified how Christian faith influenced each individuals’ mental health and coping during immigration and adjustment. This approach facilitated a more comprehensive understanding of the role of faith in Korean immigrants’ mental health, which might not be achievable using a single method alone.

**Fig. 1:**
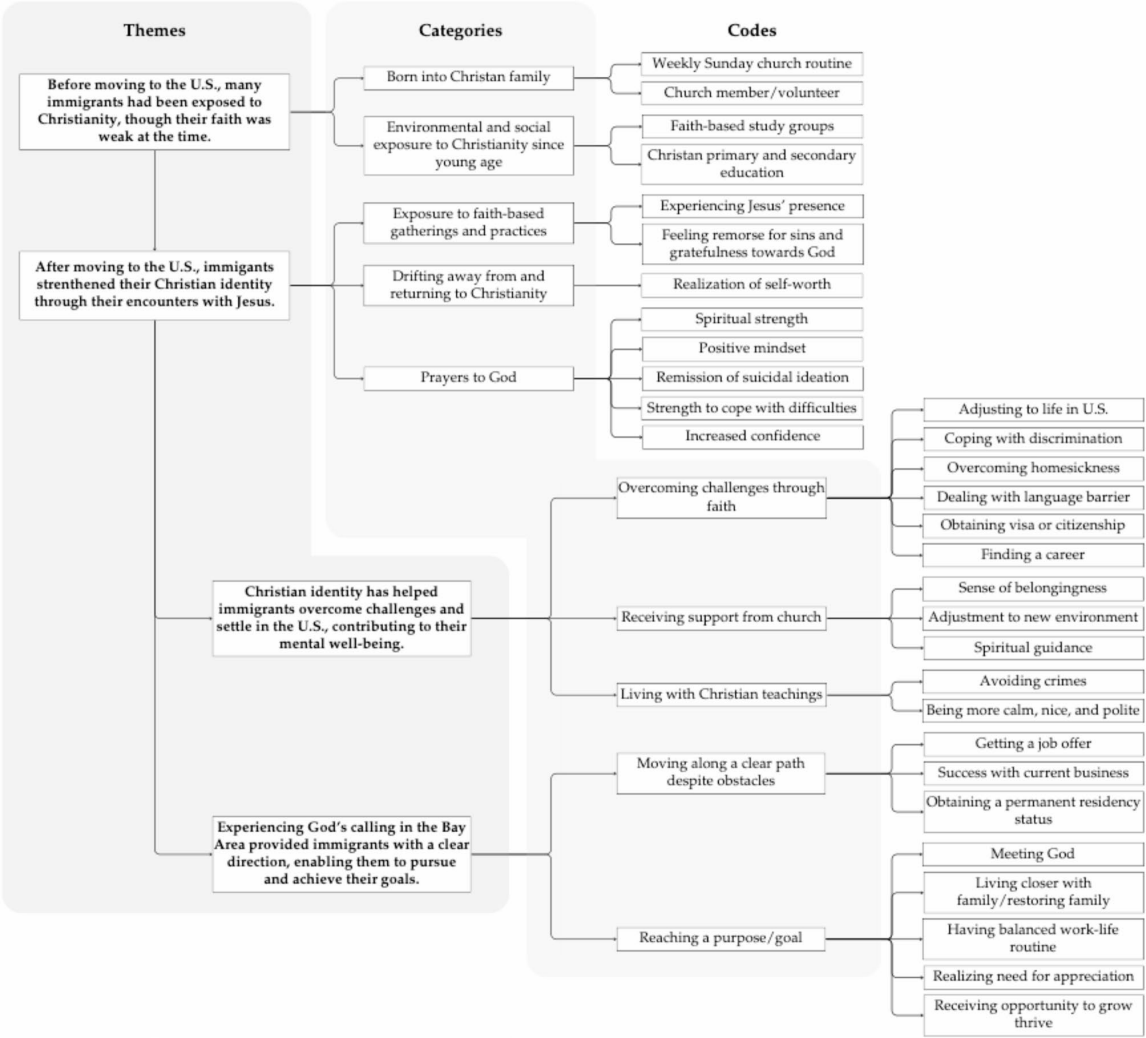
Mapping the interconnections among themes, categories, and codes in the analysis

Figure 1 depicts how faith-based identity is related to mental health during social and legal status changes among Korean immigrants, divided into four themes, and how codes are interconnected. Solid lines indicate the categorization of each theme and connection of codes.

### Participant characteristics

The inclusion criteria for eligible participants were: (1) both parents’ ethnicity is Korean, including foreign nationality; (2) adults 18 years of age or older; (3) regular attendees of a Korean church in the Bay Area; and (4) ability to read and understand English surveys and interviews. The first half of the online Qualtrics survey focused on participants’ general demographic information including gender, age, level of education, place of employment, and household income. The detailed demographic characteristics of the study participants can be found in Table 1. In the second half of the survey, participants were asked questions about their faith, spirituality, and religious faith. According to the demographic survey, the most common denomination was Presbyterian (5/22, 22.72%), followed by Southern Baptist Convention (3/22, 13.63%), American Baptist Churches USA (2/22, 9.09%), and Methodist (1/22, 4.55%). The remaining participants identified with other unspecified Baptist denominations (3/22, 13.63%) or were unsure of their denomination (8/22, 36.36%). One of the questions was about personal beliefs about God, in which the majority of the participants responded that they had no doubts that God exists (19/24, 79.17%) while a few believed in God but had some doubts (5/24, 20.83%). In terms of the level of faith, many participants believed that they were very religious (11/24, 45.83%). Many others responded that they were moderately religious (10/24, 41.67%), a participant was slightly religious (1/24, 4.17%), and a participant was not religious (1/24, 4.17%). The remaining participant chose the option ‘do not know’ (1/24, 4.17%). Regarding the level of spirituality, some participants believed that they are very spiritual (6/24, 25%), half were moderately spiritual (12/24, 50%), a few were slightly spiritual (4/24, 16.67%), and a couple of participants did not know their level of spirituality (2/24, 8.33%). We found that in terms of participants’ frequency of religious services attendance, half attended several times a week (12/24, 50%). Many others attended once a week (9/24, 37.5%), a couple attended 2–3 times within a month (2/24, 8.33%), and a participant attended several times a month (4.17%).

**Table 1:** Demographic characteristics of participants

	*N *(percent)
Gender	
Male	12 (50)
Female	12 (50)
Age (in years)	
20–30	5 (20.83)
31–40	13 (54.17)
41–50	6 (25)
Living location	
Large city or small city/town	7 (29.17)
Suburb near a large city	16 (66.67)
Do not know	1 (4.17)
Highest level of education	
Two-year associate degree from a college, university, or community college	1 (4.35)
Four-year bachelor’s degree from a college or university	8 (34.78)
Some postgraduate or professional schooling after graduating college, but no postgraduate degree	1 (4.35)
Postgraduate or professional degree including master’s, doctorate, medical, or law degree	12 (52.17)
Place of employment	
A for-profit private company, business, or individual	19 (82.61)
Other (non-profit, educational institution, self-employed)	3 (13.04)
Not currently employed	1 (4.35)
Total household income	
$35,001 - $50,000	2 (8.33)
$50,001 - $150,000	5 (20.83)
$150,001 or more	13 (54.17)
Do not wish to answer	4 (16.67)
Living alone	
Yes	11 (45.83)
No	13 (54.17)
Marital status	
Single/Never been married	20 (83.33)
Married	3 (12.5)
Separated/Divorced/Widowed	1 (4.17)
Having children	
Yes	2 (8.33)
No	22 (91.67)
Personal beliefs about God	
I have no doubts that God exists	19 (79.17)
I believe in God, but with some doubts	5 (20.83)
Level of faith	
Very religious	11 (47.83)
Moderately religious	10 (43.48)
Slightly religious	1 (4.35)
Not religious	1 (4.35)
Do not know	1 (4.35)
Level of spirituality	
Very spiritual	6 (25)
Moderately spiritual	12 (50)
Slightly spiritual	4 (16.67)
Do not know	2 (8.33)
Change in faith over the past three years	
More religious	14 (63.64)
Stayed about the same	5 (22.73)
Less religious	3 (13.64)
Frequency of attending religious services	
Several times a week	12 (50)
About once a week	9 (37.5)
2–3 times a month	2 (8.33)
Several times a month	1 (4.17)
Average length of prayers	
Half an hour	4 (16.67)
Several minutes	11 (45.83)
A minute or two	8 (33.33)
A few seconds	1 (4.17)

### Data collection and instrument

We recruited participants using purposive and snowball sampling methods at a Korean Protestant Christian church located in the Bay Area. A total of 24 Korean adults were recruited by first contacting pastors and small group leaders to inquire about distributing the invitation to church members, and by asking interviewees to refer other eligible potential participants. Those who expressed willingness or interest in participating were then sent a brief explanation of the study and possible interview dates via email, which was the primary method used to distribute the official interview invitations and interview reminders.

We focused on seven questions related to the purpose of this study as follows:

1. Tell me when and how you got to know Jesus. Did you believe Jesus Christ before coming to the U.S.?
2. How has your belief in Christianity helped you to prepare to move to/stay in the U.S.?
3. Do you feel God has clearly told you the reason of being in the Bay Area? What is your calling in this area?
4. How was your relationship with Jesus Christ when you experienced prejudice or discrimination?
5. How was your relationship with Jesus Christ during the process of gaining, or changing legal status (e.g., Korean to any visa, F1 visa to H1B, H1B to permanent residency, H1B to different H1B, or H1B has been transferred to current company)?
6. Overall, how has your belief in Christianity helped you overcome any challenges and difficulties of living in the US?
7. How has your current church in the US supported you to settle in this area?

### Interview setting

The church participants regularly attended granted permission to utilize its facility for interviews. However, there was no obligation to conduct the interviews at the church. Participants were given interview site options such as within the church premises (22/24, 91.67%), or remotely at their convenience (2/24, 8.33%). Online interviews were conducted via Zoom, a video teleconferencing software program. Regardless of interview location, all 24 interviews were audio-recorded using at least 2 voice recorders as back-ups. All interviews were conducted in English with some occasions in which participants mentioned some Korean terms in their responses. Interviews lasted from 26 to 95 min, with an average of 59 min.

### Data analysis

After the completion of the interviews, we utilized Otter.ai to conduct a verbatim transcription. The computerized transcription by Otter.ai underwent two additional rounds of manual transcription to improve accuracy: first, one author listened to the entire audio recording to correct inaccuracies, and then the entire research team reviewed unclear pronunciations or expressions to finalize a total of 24 transcriptions. We then discussed participants’ responses relevant to the purpose of this study, which was to explore the challenges faced by Korean immigrants, analyze the impact of Christian identity on their lives, and discuss the importance of spiritual health on their psychological well-being. For privacy purposes, we used ‘Participant #’ in place of participants’ names. To present the qualitative findings clearly and concisely, we selected a limited number of quotes that best exemplified each code within the themes rather than including all participant responses. This approach allowed us to highlight the patterns and shared experiences that emerged across multiple participants, while maintaining focus and minimizing repetitive data.

## Results

We collected quantitative data on participants’ belief in Christianity through an online Qualtrics survey. Likert scales were employed to measure participants’ mental health and assess their spirituality. These questions covered aspects such as the frequency of praying, reading the Bible, attending church, and participants’ views on God. Following the online survey, participants were asked about their religious background and the influence of Christianity on their lives through one-on-one, in-depth interviews. The interview questions were divided into the following four themes: (1) exposure to Christianity, (2) strengthening their belief in Christianity, (3) overcoming challenges and settling in the United States, and (4) experiencing God’s calling in the Bay Area. By combining the quantitative and qualitative data, we employed a qualitative study approach to explore the connection between the two.

### Before moving to the United States, many immigrants had been exposed to Christianity, though their faith was weak at the time

Early exposure to Christianity before coming to the United States shaped participants’ religious involvement and perspectives even when their personal faith was not yet strong. Participants who were born into Christian families described their weekly Sunday churches as a habit formed by parental reinforcement rather than personal choice. Church involvement often extended beyond just attending church services, as several participants became active members by volunteering, serving, or participating in worship groups which provided a sense of belonging and community. In addition, environmental and social factors such as attending Christian elementary schools or participating in Bible study groups further reinforced early exposure to Christianity. These experiences introduced participants to the church culture from a young age, shaping their understanding of Christianity and strengthening their development of faith later in life.

#### Born into a Christian family

##### Weekly Sunday church routine

Immigrants who were born into a Christian family often attended weekly Sunday church services. Many participants (16/24, 66.67%) mentioned that their exposure to Christianity began through regular Sunday services with their families, which became a weekly habit. Although their faith in Christianity had been weak as a child, many followed their parents to church. Several participants (3/24, 12.5%) mentioned feeling forced to attend services. Participants born into a Christian family, including Participant #2, stated that they had no personal connection or faith in God, but continued to attend church as part of their weekly routine:

It wasn’t like I was going to church because I wanted to pray.... It was more like a routine of going to church on Sundays. (Participant #2)

In addition, Participant #7 explained that he went to church primarily because his parents regularly attended Sunday services. Similar to Participant #2, his involvement with church was shaped by family routine and expectations rather than personal faith:

I was born in [a] Christian family.... I had no choice... they go to church so I had no choice. (Participant #7)

##### Church member and volunteer

Having been born into a Christian family, individuals became active church members and volunteered during weekly services. A few participants mentioned that they were involved in church activities such as singing or playing instruments in the worship groups. Furthermore, the church provided individuals with a sense of community, as participants had many connections and friends within the church. Although participants had a weak faith when they were young, participating in church services and being part of the church community gave participants a purpose to attend church every week. Participant #8 shared his experience of serving as an altar boy at his Catholic church:

My mom was a Catholic when I was growing up, so I followed my mom to the Catholic Church. In elementary school, I was an altar boy and then we moved to America. (Participant #8)

Participant #23 mentioned that serving at the weekly Sunday services helped strengthen his faith and deepen his religious commitment. By actively participating in the service as part of the music group, he experienced a sense of purpose and developed a stronger connection with the church community:

I went to church every week and I was serving. I was playing [the] violin in the service I was in every week. (Participant #23)

#### Environmental and social exposure to Christianity since young age

##### Faith-based study groups

In the Qualtrics survey, participants were asked about their personal beliefs about the Bible. The majority of participants (18/24, 75%) believed that ‘the Bible is perfectly true, but it should not be taken literally, word-for-word. We must interpret its meaning,’ followed by a few participants (4/24, 16.67%) who believed ‘the Bible means exactly what it says. It should be taken literally, word-for-word, on all subjects.’ The remaining participants selected that ‘the Bible is an ancient book of history and legends’ (1/24, 4.17%) and ‘I don’t know’ (1/24, 4.17%). Participant #2 shared that his initial encounter with Jesus came through exposure to the Bible study groups and reading Bible verses:

I was having Bible studies on a regular basis.... I learned about the Bible, about Jesus [and] the story about Jesus, all the verses.... I think [that was when] I first got to meet Him. (Participant #2)

Similarly, Participant #12 described her involvement in a prayer group, Bible classes, and church activities such as missionary work and helping with Sunday school, which she felt strengthened her faith and “filled her up”:

[I] have a prayer group.... There’s [a] Bible class [where] you get to share [in] small groups. You get to do things like missionary, help out with the Sunday school. And I think that fills you up. (Participant #12)

##### Christian primary and secondary education

Prior to moving to the United States, several participants attended a Christian school in Korea. Their parents had enrolled them in a Christian elementary school where they were exposed to the church. Participant #2 shared their experiences of learning about the story of Jesus and Bible verses at the Christian elementary school:

In Korea, I went to elementary school [and] it was a Christian school. I was exposed to church... [and] Bible studies on [a] regular basis. (Participant #2)

Similarly, Participant #7 shared similar experiences with attending a Christian high school and being exposed to Christianity as a teenager:

After I joined the Christian school and attended that high school, [we] had a service every morning, and then Fridays, late night service. (Participant #7)

### After moving to the United States, immigrants strengthened their Christian identity through their encounters with Jesus

Participants highlighted how their faith journeys after moving to the United States were shaped by exposure to religious engagement activities, the period of drifting away and returning to faith, and routine prayer practices. Participants described retreats as significant moments during their faith journey, where the environment characterized by times of worship, prayer, and spiritual strengthening facilitated a deeper sense of connection with God. These encounters were often described as emotionally transformative, with several participants expressing feeling Jesus’ presence and feeling remorse through reflection of past behaviors that were misaligned with their faith. For other individuals, distancing from Christianity brought a sense of emptiness and lack of purpose, which led to a realization of the importance of faith. Furthermore, through prayer, individuals gained strength to cope with life’s challenges, build more positive mindsets, and develop confidence in navigating their personal and professional lives.

#### Exposure to faith-based gatherings and practices

##### Experiencing Jesus’ presence

Experiencing Jesus’ presence was one of the main factors that strengthened participants’ faith in Christianity (their Christian identity) with God. Christian retreats and short-term missionary work provided participants with opportunities for self-reflection and connecting with God. Spiritual retreats offer a quiet environment for worship, prayer, and reflection, and a setting away from daily life for individuals to focus on their relationship with God [20]. Participant #4 recounted a personal and transformative encounter with Jesus that played an important role in strengthening her Christian identity and faith. During her time in the new city, she encountered challenging circumstances that were difficult to escape. She attended church every morning, wept, and prayed, ‘If God is there, please help me.’ Then she sensed the presence of God:

What an unforgettable moment. there’s a park that I go to every day because there are some pianists playing the piano every day. I was sitting there, watching, and listening them, and I just prayed, ‘God, can you play this song? And if he [(pianist)] plays, I’ll believe You.’ And then, he played it. ‘Okay, You’re there!’ I cried. He [(God)] listened to me. That moment, I think I met Jesus. (Participant #4)

In addition, Participant #20 shared his experience of feeling God’s presence during a Bible study session, which he described as an unexplainable sensation. He shared this moment, which not only affirmed his belief but also strengthened his commitment to his faith:

I experienced [a] warm, fussy feeling. I cannot really explain well about that feeling but it was a moment with Jesus. Since then, I have no doubt about believing in Jesus Christ. (Participant #20)

##### Feeling remorse for sins and gratefulness toward God

Participants reflected on their feelings of remorse for sins and gratefulness towards God during prayer sessions at Christian retreats. As participants encountered the presence of Jesus, many shared that they cried due to feeling overwhelmed with guilt as they prayed to God. Through the realization of repentance of sins and gratefulness towards God, participants strengthened their Christian beliefs. Participant #2 mentioned that during the retreat, he felt that ‘He was really there.’ Since then, he felt God had planned and ‘guided’ his future:

During my high school year, I went to retreats.... That was the first time [I] started crying when I was praying. ... I felt like I was... repenting my sins and my sinful nature and how thankful I had been. (Participant #2)

Similarly, Participant #3 shared her personal experience of encountering God for the first time during a church retreat. She was reluctant to attend a retreat, but there was a person who encouraged her to join. When she recounted that ‘He came to me,’ she also described feeling a sense of guilt for her past sins, which ultimately helped strengthen her faith:

I didn’t even know how to pray at the moment but I just kneel down and then do what I can do at the moment.... And then, that’s the time that I met Jesus. I felt Him.... I felt His hug, I feel He is holding my hand. I received the tongue[s] [(glossolalia)]. All that in one night.... I felt a little bit guilty about I met God first than other people in the retreat center.... But I’m new to Christian... then He came to me. (Participant #3)

#### Drifting away from and returning to Christianity

##### Realization of self-worth

Drifting away from Christianity led individuals to reflect on their belief, often resulting in a return to their Christian faith. Several participants emphasized experiencing a sense of emptiness when they stopped attending church services. Additionally, despite having a stable job and career, participants felt that everything was temporary and that they were ‘missing something important.’ Participant #8 shared feeling dissatisfied and lacking a sense of purpose in life as he drifted away from his faith, despite his stable life:

We moved to America and in middle school, I fell away from [the] Christian faith.... But then in the middle of high school, I felt that... having [a] temporary existence made me very empty.... I think that feeling drove me to church spiritually. (Participant #8)

In addition, Participant #13 described a moment of self-realization, explaining that through sharing God’s faith, she recognized what had been missing in her life and the significance of religion in her daily life:

Sharing God’s faith... was so nice. That’s when I realized this is what I am missing. I need this. (Participant #13)

#### Prayers to God

##### Spiritual strength

Praying to God has been a source of spiritual strength for individuals, helping them overcome self-doubts and increase their confidence. According to the Qualtrics survey responses, half of the participants (50%) strongly agreed that they pray because they believe that praying is the best way to address personal problems. Through prayer, many participants strengthened their belief in God knowing that they are not alone in their academic and career paths. They strongly believed that God was always by their side. The majority of the participants, including Participant #14, shared how praying to God had helped navigate challenges in career after moving to the United States, providing them with spiritual strength and resilience:

Even though He gives you a different answer.... The answer means He also changed your mind to accept that new answer.... I agree for that... so that’s why we have to pray every time. (Participant #14)

Participant #16 expressed the spiritual and psychological support of prayers during their immigration to the United States and their settlement in the Bay Area:

I always think that I only see small, but God sees more. So He has a better plan for me because He knows me better than myself. That always helps me. (Participant #16)

##### Positive mindset

Many Korean immigrants were able to build a positive mindset through prayers to God. Research suggests that maintaining strong attachments to God through regular prayer is associated with improvements in psychological well-being including self-esteem, optimism, and life satisfaction [21]. Survey results showed that several participants (8/24, 33.33%) strongly agreed that praying makes them better individuals. Several others (9/24, 37.5%) picked ‘agree’ to the same statement. Through their trust and belief in God, participants conveyed that they found relief from life stressors and worries. Despite encountering difficulties, they expressed resilience by shifting their mindset and looking at the positive aspects, which contributed to their mental well-being. Participant #10 shared her experience of feeling more ‘positive’ in life after she started praying to God:

After I trusted or met Jesus Christ, He gave me peace. And then things always [started] turning [in a] good way. I think more positively, [and] I don’t have to blame myself all the time. (Participant #10)

Participant #15 shared the positive impact the church had on her mental health. She explained that being part of the church community provided emotional support, a sense of belonging, and opportunities to heal during difficult times:

Mentally, [the church community] gives a lot. They support a lot. I healed a lot in this church. (Participant #15)

Additionally, Participant #16 described how her faith served as a vital source of strength, offering her confidence and an optimistic future. She expressed that even when outcomes were uncertain or beyond her control, she had confidence that God’s plan was ultimately better than her expectations:

I always had that confidence in God. Even though I [really] wanted something but it didn’t happen, I always think in a positive way. There’s something I don’t see, God has the better plan. (Participant #16)

##### Remission of suicidal ideations and other mental health issues

Participants experiencing mental instability, including thoughts of suicidal ideations, found emotional support through prayers to God. There are associations between religion and benefits for psychological distress including depression and anxiety [21]. Praying to God has helped relieve life stressors and provide immigrants with hope and mental strength. Participant #13 shared how prayer had saved her from suicidal thoughts through receiving comfort and reassurance from God:

I would go into [a] cathedral and cry there all night and pray. He saved my life from there cause I was so suicidal. But then all things work together. Looking back, everything was His grace. That’s how I met Jesus. (Participant #13)

In addition, Participant #14 shared how involvement with the church contributed to her mental stability and helped her overcome periods of depression, providing both spiritual guidance and a supportive community. She described how participating in church activities and praying offered a sense of purpose, comfort, and resilience during those challenging times:

Initially... even though someone tried to help me, I didn’t want to interact with others, but I started [to] attend a Bible study... for two years... and then I finally became okay.... [The initial] feeling came from my previous job. ... I think I had some kind of depression. (Participant #14)

##### Strength to cope with difficulties

In addition to spiritual resilience and positive mindsets, praying to God provided participants with the strength to cope with challenges. When asked how often they pray for guidance in decision-making, nearly half of the participants (10/24, 41.67%) mentioned ‘always,’ while several others (9/24, 37.5%) answered ‘most of the time.’ They believed God had guided them through their decision to move to the United States, as well as through the processes of overcoming the challenges of immigration and the difficulties of securing employment. Participant #5 mentioned that she believes every experience is a lesson intended for her improvement and that better outcomes will follow in the future:

My faith... also helps me with coping with problems that I deal with. ... It just gives me more strength to cope with the struggles I have. (Participant #5)

Participant #21 mentioned that she is currently alone and finds it difficult to trust others. However, her faith in God helped her overcome difficulties, reminding her that she is never truly alone:

Without this belief in Christianity, I would be so lonely. ... I trust in God, so I know that I’m not alone. ... It helps me to overcome difficulties and loneliness and everything. (Participant #21)

##### Increased confidence

Trusting in God helped participants have confidence in their life paths. Several participants expressed that they were able to overcome challenges with God’s presence. They described feeling no fear because they trust that God will guide them along the best paths. Even when things did not turn out as desired, they found strength in accepting it as God’s will. Participant #3 shared that she is not afraid of facing obstacles because she believes God will always help her:

[I am] not afraid to get on other challenges even though I don’t want to do [it]. I can accept it. I can go through [it] because He will make it happen and He will help me out. (Participant #3)

Likewise, Participant #23 discussed that believing in and trusting God allowed her to feel confident during her immigration to the United States. She trusted that God was guiding her according to His plan, which helped her move forward without looking back:

I believe God wants me to [go] to where I should be. God always leads me to where I belong. ... I trust staying in South Korea is not better than moving to the United States. (Participant #23).

### Christian identity has helped immigrants overcome challenges and settle in the United States, contributing to their mental well-being

Participants believed their Christian identity (faith in Christianity) has strengthened over the years, providing a crucial resource for coping with stress and uncertainty during their life transitions. In the Qualtrics survey, participants were asked about their level of belief over the past three years. The majority of participants believed they have become ‘more religious’ (14/24, 58.33%), with few believing their belief have ‘stayed about the same’ (5/24, 20.83%) or had become ‘less religious’ (3/24, 12.5%). The remaining participants did not provide an answer (2/24, 8.33%). When many participants’ faith increased, the majority also reported regularly attending church. Half of the participants (12/24, 50%) attended religious services several times a week, while many attended about once a week (9/24, 37.5%). We also asked participants about their friends’ church attendance. Many participants knew a few friends (6/24, 25%), about half (9/24, 37.5%), or most (6/24, 25%) who attended the same place of worship. The remaining three participants stated that all of their friends attended church (3/24, 12.5%).

We measured how participants might have felt or behaved due to their legal status in the United States using the Center for Epidemiologic Studies Depression Scale (CES-D) [22]. We added a tailored question to this measurement scale: ‘Please tell us how often you have/had felt this way on a weekly basis during the time period that you experienced challenges related to your legal status in the United States, such as waiting for a work permit, H1B lottery results, the U.S. Permanent Resident Card (so-called Green Card) approval, or any challenges.’ Responses to the CES-D questions were scored and summed. The CES-D classifies responses based on the total score: (a) 0–16: no to mild depressive symptomatology, (b) 16–23: moderate depressive symptomatology, and (c) 24–60: severe depressive symptomatology. Among the 24 participants, the majority were classified as having no to mild depressive symptomatology (16/24, 66.67%), with a few participants having moderate depressive symptomatology (3/24, 12.5%) and severe depressive symptomatology (3/24, 12.5%). The remaining participants (2/24, 8.33%) did not provide a response.

**Fig. 2:**
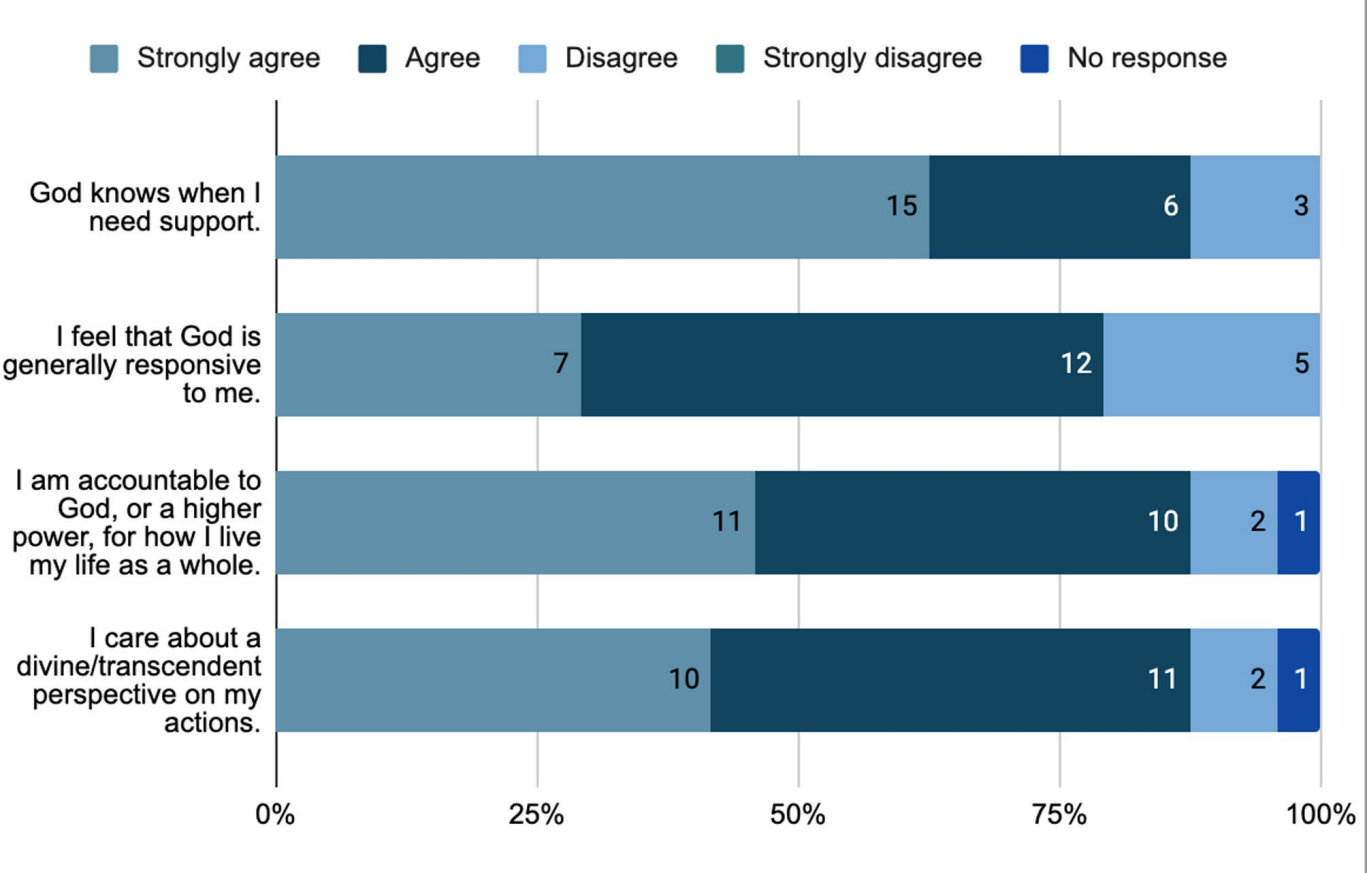
Participants’ personal understanding of God

Within the Qualtrics survey, participants were asked several questions regarding their personal understanding of God (Fig. 2). For the statement ‘God knows when I need support,’ the majority of participants selected ‘strongly agree’ (15/24, 62.5%) followed by ‘agree’ (4/24, 16.67%), and 3 participants (12.5%) disagreed. Additionally, participants rated the following sentence ‘I feel that God is generally responsive to me.’ Among the 24 participants, 7 (29.17%) strongly agreed, 12 (50%) agreed, and 5 (20.83%) disagreed.

Participants rated the extent to which they agreed or disagreed with the following statement: ‘I am accountable to God, or a higher power, for how I live my life as a whole.’ Of the 24 participants, 11 participants (45.83%) selected ‘strongly agree,’ 10 participants (41.67%) selected ‘agreed,’ 2 participants (8.33%) selected ‘disagree,’ and 1 participant (4.17%) did not answer. In addition, we asked the participants to rate the statement ‘I care about a divine/transcendent perspective on my actions.’ The majority of participants either strongly agreed (10/24, 41.67%) or agreed (11/24, 45.83%), and a few participants disagreed (2/24, 8.33%) or did not answer (1/24, 4.17%).

#### Overcoming challenges through faith

Through their Christian identity, participants described gaining emotional resilience and mental strength throughout their immigration journey. Faith served as a coping mechanism that help alleviate feelings of anxiety, depression, and isolation, particularly as they faced discrimination, language barriers, and legal transitions. Participants emphasized that their Christian faith gave them a sense of purpose in life and emotional support, which helped reduce psychological distress during challenges.

##### Adjusting to life in the United States

As Korean immigrants moved to the United States, many experienced difficulties settling into a new environment. This was especially true for those who moved to predominantly White communities, where many felt discriminated against as an ethnic minority. Participants emphasized that their Christian faith provided a meaning in life and emotional support which reduced feelings of anxiety. A strong Christian identity offered immigrants a source of connection and emotional support. Furthermore, attending Christian churches provided participants with opportunities to meet new people and build their community, supporting participants against loneliness and discrimination. Participant #1 shared her experience of having faith groups within the Christian community, which helped her adjust to life in the United States:

I think having that support form the cell group [(bible study group)] was a very important factor in helping me adjust to my life living in the Bay Area. (Participant #1)

Additionally, Participant #8 mentioned that his faith in Christianity provided him with a new perspective in life, enabling him to overcome the challenges of being an immigrant:

When I had a different perspective in life, that provided motivation for doing everything. It’s not just about adjusting to life in America, but living life fundamentally. Part of that is adjusting to whatever difficulties I faced as an immigrant. (Participant #8)

##### Coping with discrimination

Immigrants often encounter mistreatment as they struggle to fit into the new community [23]. In predominantly white communities, participants were particularly vulnerable to facing workplace discrimination through verbal assaults. Through their faith in Christianity, participants were able to have resilience and maintain mental stability. Additionally, the church provided individuals with a supportive community and their prayers to God helped guide them during those difficult times. Participant #4 shared her experience of overcoming abuse through Christianity:

I went through a lot of things like abuse. People tried to abuse me but the church made me more strong, and I relied on Jesus whenever I had a hardship. That’s how I got through [with] Christianity. (Participant #4)

In addition, Participant #21 shared her experience of discrimination at her boarding school where she was considered the minority population. She decided to attend a boarding school for enhanced education and improved college readiness. While socializing with Asian boarders, who constituted a significant segment of student groups, she was subjected to hostile comments that demeaned her race and ethnicity. Nonetheless, to manage the situation, she dismissed it and moved on:

We pass[ed] by some group of White students [who] would be like ‘Go, go back to your country!’ ... They scream[ed] [and] yell[ed] to us. (Participant #21)

##### Overcoming homesickness

After leaving their home country, Korea, many participants expressed their feelings of homesickness. Participants struggled to adapt to life in the United States, adjusting to a new environment and facing challenges with language barriers and acculturation. They often felt lonely due to not being able to connect with friends and relatives back in Korea. Participant #5 described missing her home country, however, she noted that her faith, believing that God has a clear plan for her to stay in the United States, helped her cope and maintain mental stability:

When I moved here, I was struggling because I missed everyone in Korea. I was like, ‘Is this the right path? Why did God send me here?’ And then I always thought, ‘Okay, He sent me here because He has some plans for me,’ not in Korea but here. So I think just believing that He had plans for me, and He wouldn’t have sent me here unless He had big plans for me. (Participant #5)

In addition, Participant #13 mentioned that reading the Bible and praying to God empowered her to stay strong and overcome homesickness. Her faith allowed her to recognize and appreciate the advantages of living in the United States:

I spent a lot of time studying [the] Bible and praying. That keeps me strong. Otherwise, I’m depressed... lonely... [and] homesick. But because I hold on to [the] Bible, it makes me less lonely. (Participant #13)

##### Dealing with language barrier

Overcoming the language barrier is one of the significant struggles for immigrants [24]. Despite several participants having acquired English proficiency prior to moving to the United States, many still encountered difficulties in speaking English fluently. Nonetheless, participants were able to continue their journey through their trust in God that coming to the United States was part of God’s plan for them. Participant #5 explained that her family advised her on how to avoid social marginalization, such as attaining financial stability in this foreign country, maintaining a respectable appearance through appropriate clothing, and demonstrating fluency in English. She further noted that overcoming the language barrier was particularly critical in mitigating the risk of being demeaned by others. She highlighted the necessity of self-advocacy, stressing the value of articulating her needs and viewpoints across multiple contexts using English:

It is sad when you’re in a country that you don’t even speak fluent English. And then you are looked down upon. That is sad. My sister basically told me, if you don’t understand something, just ask because if you stay quiet, then they would misunderstand you. You have to be speaking up for yourself and everything. (Participant #5)

Similarly, Participant #23 reflected on the challenges of acquiring English proficiency after immigrating to the United States. He emphasized that, despite these difficulties, his faith provided a sense of direction and belonging, as he believed God continually guided him to where he should be. He illustrated this struggle by noting the variation in regional speech across the country:

I believe God, He wants me to [be] where I should be. God always leads me to where I belong.... Even the Western people and people in the East of the United States, they speak differently. One of my colleagues is from Michigan and I don’t understand him at all. (Participant #23)

##### Obtaining a visa or citizenship

**Fig. 3:**
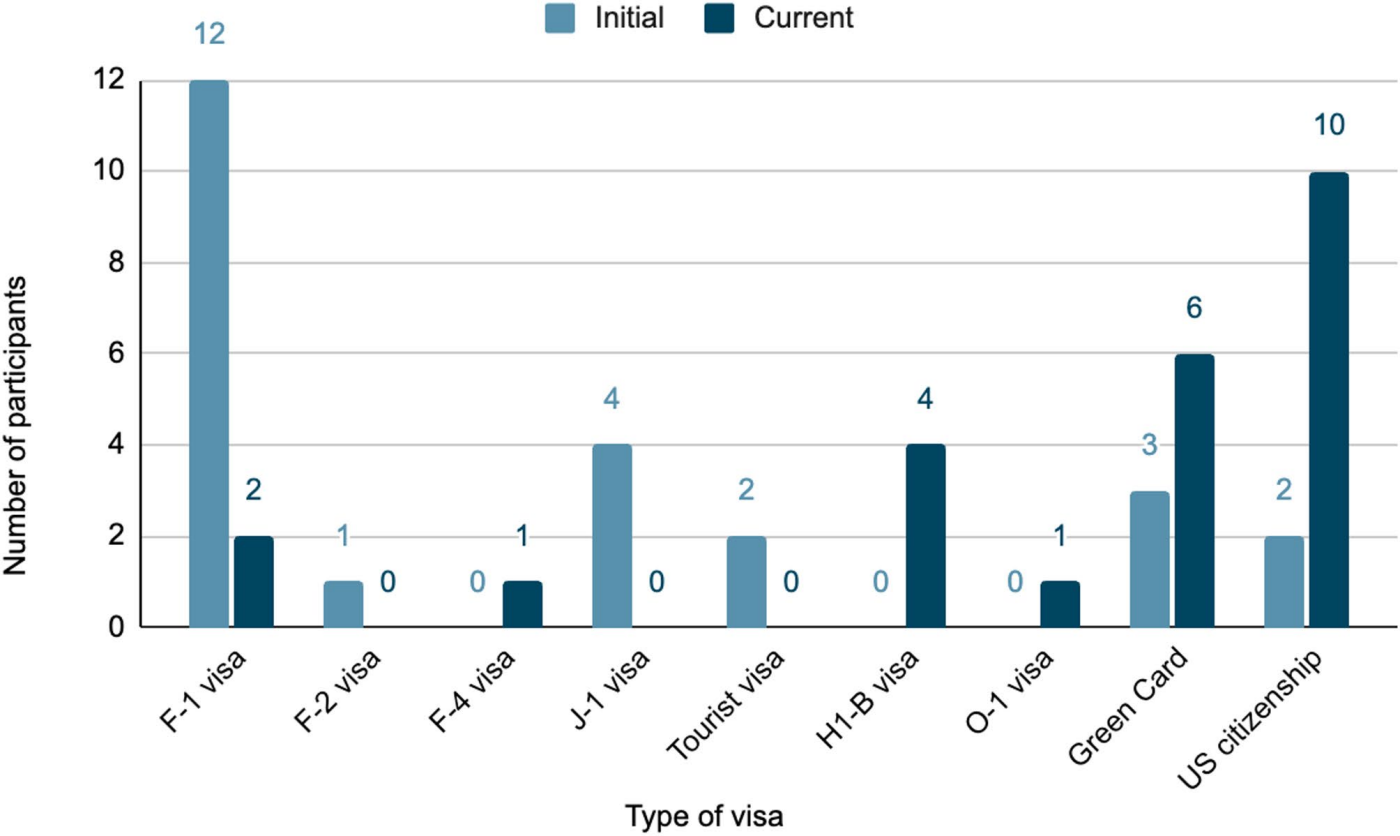
Participants’ initial and current visa status

Our Qualtrics survey results indicate that half of the participants came to the United States on an F-1 student visa (12/24, 50%) (Fig. 3). Several participants arrived with a J-1 visa (4/24, 16.67%), a tourist visa (2/24, 8.33%), or an F-2 visa (1/24, 4.17%). Additionally, some participants already had a Green Card (3/24, 12.5%) or were born in the United States (2/24, 8.33%). Currently, many participants hold either a U.S. citizenship (10/24, 41.67%), Green Card (6/24, 25%), or H1-B working visa (4/24). A few participants still have an F-1 visa (2/24, 8.33%), while others have an F-4 visa (1/24, 4.17%) or O-1 visa (1/24, 4.17%) (Fig. 3).

Participants stated that their belief in Christianity had helped them overcome challenges in obtaining their legal status. In the survey, participants were asked to rate the statement ‘My belief in Christianity had/has helped me a lot to overcome challenging times to get my stable legal status in the United States.’ The majority of participants strongly agreed (12/24, 50%) or agreed (4/24, 16.67%), while several participants felt neutral (6/24, 25%) or disagreed (2/24, 8.33%) with the statement.

International students on F-1 visas are only allowed to stay in the United States temporarily as long as they are enrolled in an academic or language training program [25]. Half of the study participants (12/24, 50%) came to the States with an F-1 visa, and Participant #21 is one of them. Upon the completion of an educational program, individuals on F-1 visas are required to transfer to another school, change their educational level, or apply for a different visa status if they would like to stay. Otherwise, they are given 60 days to leave the country [26]. As immigrants faced the challenges of maintaining their legal status, Participant #21 expressed her gratitude for Jesus who helped her through her struggles:

I was thanking Jesus... because I had a mindset [that] things like visa, that’s something outside of my control. If I don’t get a valid visa, I would have to leave the country in the next few years. [If so] then I thought that was Jesus’s calling. Somehow I got selected so I thought maybe He wants me to stay here. (Participant #21)

The immigration rate in the United States has been lowered to 3% for more than a century as a result of the Emergency Quota Law in 1921 [27]. Despite the high number of Green Card applications, the majority of the applicants are unlikely to receive them [28]. Additionally, the application process may span approximately 3 years and it generally takes 2 years for it to become available [29, 30]. Overall, there is a low chance of getting a Green Card and the process takes years. Participant #10 expressed her belief in God’s help throughout the quick and smooth immigration process:

Me and my son, we got the Green Card. [It was] so quick because usually, I heard that you wait. When I applied, four months [after], he got it. And then three months later, I got my Green Card. So everything was very smooth and I strongly feel that is God’s help (Participant #10).

##### Finding a career

The majority of participants migrated to the United States with the purpose of finding job opportunities and pursuing their career in the United States. Within the Qualtrics survey, more than half of the participants (14/24, 58.33%) strongly agreed or agreed to the statement ‘I would like to stay in the United States as long as I can.’ Several participants felt neutral (9/24, 37.5%) or disagreed (1/24, 4.17%). When applying for jobs, immigrants must acquire an H-1B work visa, which allows them a temporary working permit [31]. Obtaining a working visa involves a competitive process through entering a visa lottery or receiving company sponsorship [31]. Participants shared that they were able to overcome the challenges of finding a career through their prayers to God and their belief in Christianity. Participant #16 shared how trusting in God allowed her to secure the career she wanted. She explained that praying to God and devoting herself as a Christian helped her navigate through the competitive job market and ultimately achieve her dream job:

As a Christian, I know it’s not about me because God always showed me... and led me to the best way. I trusted in Him and devoted myself as a 100% follower. So I got a job. I got promoted as an Asian marketing host. (Participant #16)

Participant #18 mentioned that God had guided him toward a career that aligned well with his personality. Regardless of the location, he believed that God was working through him and was willing to work wherever he could.

I think it’s also providence that God helped me find a career that really well suits my personality. (Participant #18)

#### Receiving support from the church

##### Sense of belongingness

Ethnic churches play a crucial role in helping immigrants navigate the new environment. They foster a sense of belonging and community [32]. As indicated by the inclusion criteria of this study, all 24 participants were regular attendees of a church. Many of them emphasized the positive environment of the church and the goodness of God as presented by the community. Participant #5 shared how she felt a powerful sense of comfort at her church, as it made her feel she was not alone even during difficult moments:

I never knew the power and comfort of other people saying, ‘I’ll pray for you.’ But now, certainly I believe in the power of prayer and it’s a lot of comfort and relief for me because I have experienced and I do know the power of prayers and... the fact that we can share the struggles. (Participant #5)

Participant #6 shared his belief that the church is filled with people who are willing to support and pray for others. Within this church group, people can feel a strong sense of belonging with others who share the same values, and they can make a positive impact by serving the community around them:

[The] house of worship is made of people. I think it’s important to have that sense of belonging.... I think it’s equally important to go outwards for missions and serve the community... who needs a prayer or whatever means of support that they need. (Participant #6)

##### Adjustment to a new environment

Korean immigrants facing challenges in a foreign country often build strong connections by participating in Korean church services and activities to overcome the difficulties of adaptation [33]. Many participants stated that the church is a welcoming community and with that, they felt a sense of belonging. This sense of belonging stems from the social support received from the church. Participant #9 specifically expressed how the church and the religious community have positively impacted him:

Wherever I go, a community in church is very accepting. There’s a community that you can belong to. A sense of being centered around a certain belief that helps me get through life. (Participant #9)

Similarly, Participant #11 stated that, as an immigrant, she relies on God for support in all aspects of her life and has no expectations of help from anyone other than God. She shared how the church helped her settle in the Bay Area, providing a welcoming community as she navigated a new environment:

I think my community... this church is my primary community so this church definitely helped me settle in this area.... Just being able to come to this community and pray for each other [and] building this fellowship with one another. I think that itself had a lot of impact on how I could settle here. (Participant #11)

##### Spiritual guidance

A healthy church community forms the basis of spiritual and emotional development [34]. Spiritual health is one of the six dimensions of wellness and it involves faiths, beliefs, values, and morals [35, 36]. Benefits such as finding peace, compassion, and fulfillment are commonly associated with spiritual wellness and may help individuals overcome challenges [36]. Some participants mentioned that church has positively impacted their spiritual health. Participant #11 emphasized how her Christian faith provided her with spiritual grounding and guidance as she adjusted to life in the United States:

As an immigrant and living in a country where I didn’t grow up... I’m not familiar with anything that’s going on here. I just have to lean on God for every single thing because I just don’t have any expectation or knowledge or anything that can help me but God. So definitely, in every aspect is God. (Participant #11)

Before moving to the Bay Area, Participant #17 attended a church in a different city, where he experienced struggles with little spiritual support. He felt sufficiently spiritually supported by the current church and emphasized the importance of having a healthy community at church:

This church is pretty healthy compared to other churches, and when church [is] healthy, it... give[s] you more spiritual support. (Participant #17)

#### Living with Christian teachings

##### Avoiding crimes

Adults with strong religious beliefs are more likely to be healthier, while individuals who do not hold such beliefs are more likely to binge drink and use drugs [37]. A study has shown that religion and spirituality have a huge influence on substance use prevention and abstinence [38]. Participant #7 highlighted the impact of religious beliefs on health, specifically health behavior:

If I don’t have the religion, then I might [turn to] alcohol, like drugs, or something fun, like partying [to] try to fill that emptiness. I live by myself, but at least God is with me. (Participant #7)

In addition, Participant #9 shared how religion has shaped his personality and provided a guideline on how to be a “good person.” This would enable him to make a positive impact on our society by practicing Christianity and showing love to neighbors around him:

If you look at Christianity, we’re taught that we should love our neighbors. Without that belief in Christianity... a lot of people won’t have that kind of a guideline to being a good person in society. Personally, it helped me to grasp a central point. (Participant #9)

##### Being more calm, nice, and polite

In Christianity, it is believed that the Bible provides truths to inspire and support individuals in their pursuit of a righteous mindset. People who believe in Christianity generally believe that God offers the opportunity to bring blessings to others through a positive demeanor [39]. In addition to participants’ thoughts on the impact of living with Christian teachings on avoiding crimes, Participant #12 mentioned that, with the help of Christianity, she was better able to stay positive:

My relationship [with] Jesus made me more calm. I think I’m nicer to situations and people. If I didn’t know Jesus, I would have been angrier, more aggressive, more rude. But I think I’m the opposite—calm, nice, p olite. (Participant #12)

In addition, Participant #15 described how strengthening her belief in Christianity helped change her view towards problems and she trusted that God will lead her to the best path. She shared how before we were Christian, she would say bad words and blame God when things did not go according to her. However, she was now able to think of situations positively:

When I had some problems before I [was] too serious with God, I thought very bad words.... I said just bad words... to God like ‘Are you happy?’ ... But now, [I think] maybe He has things for me.... He will give me the more good way than my situation now. (Participant #15)

### Experiencing God’s calling in the Bay Area provided immigrants with a clear direction, enabling them to pursue and achieve their goals

**Fig. 4:**
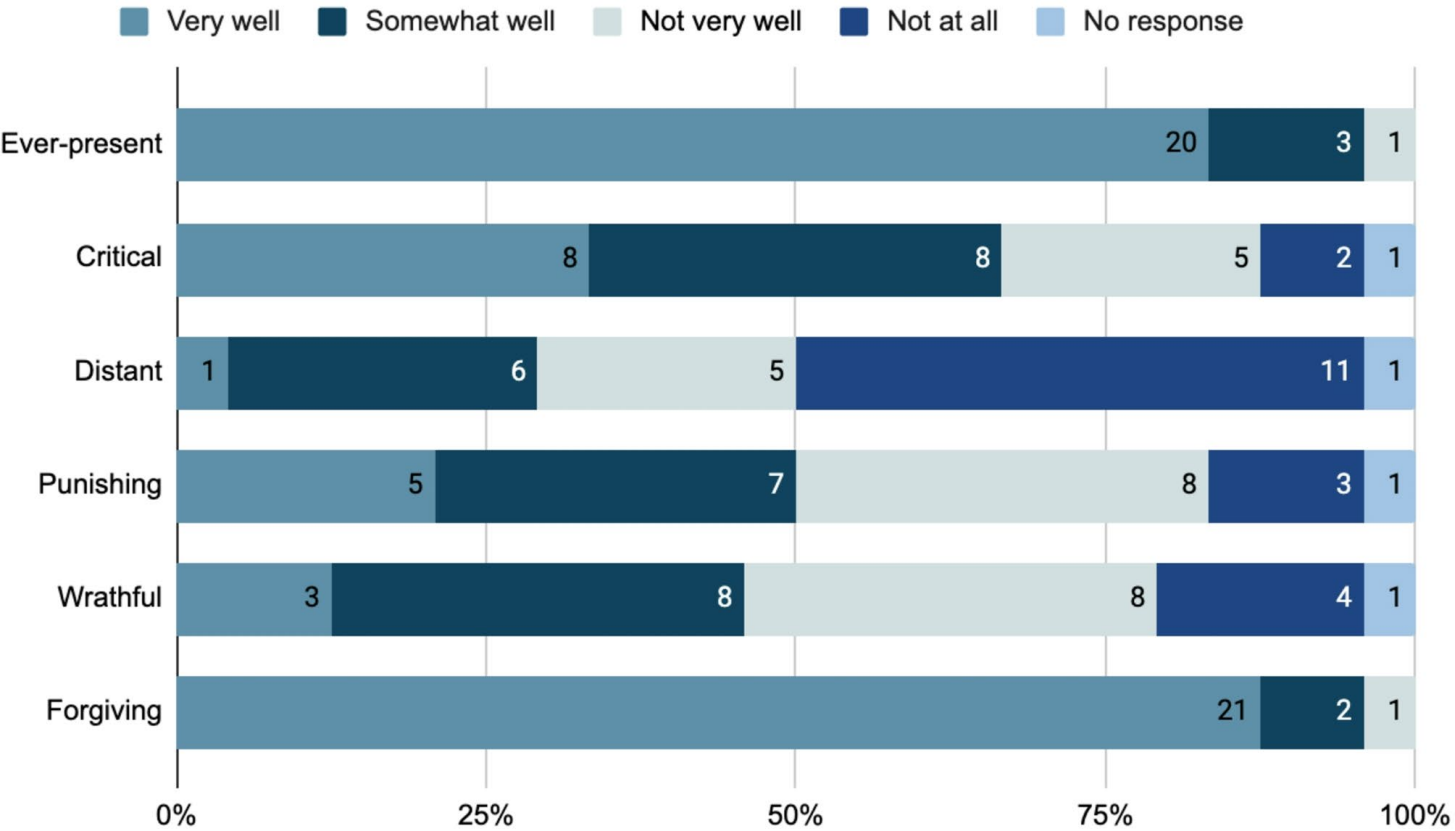
Participants’ perceptions of how various words describe God

In the quantitative survey, participants were asked how well they believe the following words describe God: ever-present, critical, distant, punishing, wrathful, and forgiving (Fig. 4). The majority of participants felt the word ‘ever-present’ (20/24, 83.33%) and ‘forgiving’ (21/24, 87.5%) describes God ‘very well’ For the word ‘critical,’ two-thirds of participants selected ‘very well’ or ‘somewhat well’ (16/24, 66.67%), while two-thirds chose ‘not at all’ or ‘not very well’ (16/24, 66.67%) for the word ‘distant.’ Responses were relatively evenly split for the word ‘punishing’ (‘not at all’ or ‘not very well’: 11/24, 45.83%; ‘very well’ or ‘somewhat well’: 12/24, 50%) and ‘wrathful’ (‘not at all’ or ‘not very well’: 12/24, 50%; ‘very well’ or ‘somewhat well’: 11/24, 45.83%) as descriptions of God.

#### Moving along a clear path despite obstacles

##### Getting a job offer

A participant believed that religion influences where one lives. A few participants shared their unique experiences with God’s calling. Participant #1 who has been living in the Bay Area said that if God tells her family to move somewhere else, she would go whether she likes it or not, regardless of her readiness to move. Likewise, when asked if she ever felt that God had clearly told her the reason to come to the Bay Area, she answered without hesitation that she came here because of God’s calling through her husband:

It was very clear because my husband got laid off. On the day of the announcement, he got a call from a company here saying [they] are offering this job. My husband tried hard to apply to other places in San Diego, but he didn’t hear back. We had no choice but to come here. It was very clear. We didn’t have to decide. (Participant #1)

In addition, Participant #2 shared his experience of how God led him to the career he has now. He explained that he had not originally planned to be in California, but when the only job offer he received was in the Bay Area, he believed God had guided him there for a reason:

I never expected myself to be in California, I wanted to go to New York. But somehow my jobs weren’t working out that way, and I just got one offer here so I ended up being here. ... I thought about how God... routed me to this path for me to be here. (Participant #2)

##### Success with current business

Based on the demographic characteristics of this study, 3 participants (3/24, 12.5%) were self-employed. One of the self-employed participants, Participant #9 who was a business owner, mentioned that he experienced God’s calling through his work. In his interview, he shared his life story, detailing how he has been to different places throughout his life such as moving to a different city back in South Korea, coming to the United States and staying in Chicago, attending to school on the East Coast, studying abroad in China, taking some time off in Japan, spending some time in Texas, and finally settling in the Bay Area. Participant #9 expressed that his life experience has prepared him well for God’s calling which is his business:

I think my current business is my calling.... I came to the Bay Area and everything aligned really great.... Through the business, my eventual goal in life is to give back to others and create value in the world.... I feel like everything that I did up to now prepared me and set me off. (Participant #9)

Participant #16 shared and emphasized how her success in managing work was due to God’s guidance and support. She had the opportunity to work during her student’s life, which allowed her to support herself financially and cover living expenses, as well as gaining experience in advance, working in the field of her business area:

With my student visa, I still got a permit to work during the summer, I got a job at the hotel.... So I got a job, I was working, I was studying. (Participant #16)

##### Obtaining a permanent residency status

South Korea is among the top five countries of origin of international students in the United States. Additionally, popular majors among international students are tech-related, business, and economics [40]. Participant #4, whose major was related to child and adolescent development, mentioned the limited job opportunities within her field. Consequently, she thought it would be difficult to obtain a Green Card after her studies. However, she did, and she believed it was God’s calling:

He [(God)] keeps telling [me] that I should stay here. I think that’s why He gave me the Green Card. Usually international students don’t like [my] major because [there are] no good jobs here. Engineer or any tech field can get an H-1B or Green Card easier than my field. So I had no expectations to get a Green Card with my major. (Participant #4)

Participant #22 reflected on how he regreted not relying on God during his process of obtaining a Green Card as he believed that the process would have gone more smoother if he had spent more time praying. When a mistake was found in his initial Green Card application submitted by the law firm he used, he had two choices, either go with that application, or withdraw it and apply again. He shared his feeling at that time:

I got panic.... I never experienced that kind of breaking down. [After] I went through a difficult couple [of] months, and I decided to re-apply. The process was pretty painful.... If I prayed and fully relied on Jesus, maybe I was able to go through it easily [and] obtaining Green Card earlier. (Participant #22)

#### Reaching a purpose and goal

##### Meeting God

Many of the study participants grew up in Christian families, but Participant #3’s family practiced Catholicism. Although she was already familiar with Jesus and Christianity in Korea, she never had a personal relationship with Him because ‘life was too busy.’ However, after moving to the United States and struggling to adjust to the environment, build new connections, and start a new life, she attended a Christian church in America. At a retreat, she kneeled and, for the first time, met Jesus, feeling His presence through a hug. As this was her first encounter with Jesus, Participant #3 expressed that this experience gave her a sense of purpose for moving to the United States:

I feel like God moved me from Korea to the State to meet Him. (Participant #3)

Participant #5 described questioning why God had sent her to the United States, but believing that He had plans for her provided reassurance and helped her feel confident about living in the Bay Area:

I was like, ‘Is this the right path? Why did God send me here?’ Then I always thought, ‘Okay, He sent me here because He has some plans for me.’... I think believing that He had plans for me, and He wouldn’t have sent me here unless He had big plans for me reassured that I can live here. (Participant #5)

##### Living closer with family and restoring family

Several participants came to the United States with their families, including siblings. Participant #5 and her sister both lived in the country but in different states. Participant #5 initially wanted to move to the East Coast, but she felt God’s calling to relocate to the Bay Area after receiving a phone call offering her a job there without her having actively sought one. She moved to the Bay Area, while her sister relocated to Seattle. Participant #5 felt this was a sign from God to live closer to her family:

The fact that I wasn’t looking for a job, and [a] big company called me is a sign from God to move me there. Because that also coincides with when my sister moved from Philadelphia to Seattle. I was like, ‘Okay, my God is leading me to be closer to my family or my sister.’ So I took that opportunity. (Participant #5)

Participant #10 shared how God had restored her family after her husband initially left her, describing how she believed God had guided her husband back:

[God] restored our family. I never thought about marrying my husband because... he abandoned me when I got pregnant. He was in Korea and when he noticed that I got pregnant, he just left... but [later] he [started] emailing me, ‘How are you?’ ... I know... everything is God... changing him. (Participant #10)

##### Having a balanced work-life routine

Multiple participants discussed their working experience in the United States and shared their stories about changing jobs for various reasons. Despite working at a big company in the Bay Area, Participant #5 faced a challenge at her workplace. After much crying and praying, she left the company for a position with a better work-life balance. She later felt she understood God’s intention behind the challenges she faced at her previous job:

My boss was really difficult to deal with. I always cried and prayed [to] God, ‘Why did you send me here? Do you really hate me?’ Eventually, I quit, and I got a job that [has a] more balanced work-life. It’s giving me more time to study and read and do whatever I want to do. So now, I’m finally seeing the reason. (Participant #5)

Participant #16 explained how changing jobs allowed her more flexibility to attend church regularly and become more socially involved in her community, providing her a structured routine regardless of the city she lived in:

I moved to New York so I was there for a year.... Then I got a job... but New York was not my city. So after a year, I was kind of missing... Vegas because it’s about people.... But I just go to church, and I go to work. For me, [it] didn’t matter [if I were] in New York, San Francisco, or in Vegas... because I just go to work and church. (Participant #16)

##### Realizing the need for appreciation

Furthermore, in Participant #5’s story about her work journey, she later shared three reasons why she strongly believed that God had planned everything she went through in her life in America. The reasons were: she moved to the Bay Area, she created a deeper bond with her family by living closer to her sister, and she switched to a different job that offered a better work-life balance. Participant #5 expressed that her new job had a positive impact on her health:

I went through struggles. That’s why I was able to get a job that’s more relaxed for me right now. It’s definitely helping my mental health and my physical health as well.... I don’t think God led me here for no reason. (Participant #5)

Participant #21 also shared her appreciation for God during the process of transitioning her legal status. She described how she relied on prayer and faith to overcome challenges that were beyond her control and believed the positive outcomes were signs from God:

I was thanking Jesus... because I had more of a mindset that things like visa... is something out of my control. If I would have to leave the country in the next few years, then I thought that was Jesus’ calling... but then I saw I got selected, so I thought maybe He wants me to stay here. (Participant #21)

##### Receiving the opportunity to grow and thrive

A few participants had previously lived in another country before coming to the United States, and Participant #12 was one of them. She had spent some time in Indonesia and enjoyed her life there, wanting to stay. However, she eventually moved to the United States, where life became challenging. Despite the hardships she faced in America, she expressed her belief that God made her move to provide her with the opportunity to grow and thrive:

I really want to live in Indonesia, but I think God put me here to work on myself. I wanted to stay there.... Life in Indonesia is great. It’s perfect. It’s very relaxing, no stress, everything’s all good. Somehow I came back, and life is hectic here. But I think if I was in Indonesia, I would have been [in the] same place. I feel when I’m here, I’m growing. (Participant #12)

Similar to Participant #12, who believed that God led her to the United States to help her grow, Participant #19 shared his thoughts on the areas in which God had helped him grow. Later in his interview, he mentioned that he preferred Southern California over the Bay Area in terms of location, but he found the people in the Bay Area interesting because they strive to do their best in everything. This made him more intrigued to stay in the Bay Area. When asked whether he felt there was a reason why God had brought him to the Bay Area, he expressed that God had led him to grow both in his career and spirituality:

I thought He wanted me to move here, so I can grow in terms of my career.... My career grew, but I think most importantly... my spirituality grew the most. So I think He wanted me to grow as His... disciple. (Participant #19)

In addition, Participant #24, who is a college student, initially thought that God put him to a place where he felt isolated from everything. Later, however, he began to think about what he truly needed to do and realized that God had sent him to the Bay Area to pursue his dream job as a physical therapist:

I want to help in a mission trip. That’s why I decided to do physical therapy. I think that’s the reason why He sent me here. [In this area, there is] a school [that] has one of the best physical therapy programs... that’s why He sent me here. (Participant #24)

## Discussion

Struggles among immigrants often center around legal status, racism and discrimination, language barriers, and other difficulties associated with adjusting to American life. Previous research studies focused on evaluating the impact of immigrants’ challenges on their mental health [41–43]. However, aspects of health including spirituality and community could provide a deeper understanding of how immigrants overcome challenges and cope with difficulties. Thus, this study used both quantitative and qualitative research methods to explore the role of religion and/or spirituality among Korean immigrants and its impact on their mental health and psychological well-being. Although this study involved a relatively small sample, all participants believed that strong religious belief in Christianity has positively impacted their mental health.

Aligned with Pargament’s (1997) theory of religious coping, participants’ reliance on prayer, spiritual interpretation of life events, and emotional support from church communities provided a framework for understanding how individuals use religious relief and community to navigate life stressors. These participants primarily demonstrated positive religious coping, where they developed resilience and overcame emotional and social challenges through strengthening their faith and building church relationships. Specifically, participants’ reflections reveal the relationship between religion and health, emphasizing how their encounter with the Christian community in the United States has strengthened their Christian identity. The connections among participants’ responses reveal a dynamic process in which religious practices translated into personal transformation, coping strategies, and life outcomes.

Figure 1 illustrates several key connections among the themes and codes that emerged from participants’ responses. Routine engagement in church services, service roles, and Bible study groups fostered a sense of belonging, spiritual guidance, and opportunities for growth, which reinforced experiences of Jesus’ presence and gratitude toward God. These spiritual encounters were closely related to realizations of self-worth, increased confidence, and the strength to cope with challenges such as homesickness, abuse, and language barriers. Likewise, as participants strengthened their faith and spiritual resilience, they reported reduced suicidal ideations, thus leading to overall better mental health. At various points during their immigrant journey in the United States, participants felt a calling from God to move to the Bay Area. They realized that after moving and settling there, some aspects of their lives improved. These improvements included having a clearer career path, receiving job offers, achieving business success, obtaining legal status, meeting their goals or purposes through spiritual growth, living closer to family, achieving a better work-life balance, and accessing better opportunities. These connections highlight how Christian identity served as a multidimensional source that connected psychological well-being and adaptation to life in the United States.

In addition to the benefits of strengthened Christian belief, Korean immigrants developed positive traits. Participants reported acquiring traits such as having a positive mindset, increased confidence, and developed compassion. From the one-on-one, in-depth interviews, we gathered a collective response suggesting that Christianity has helped Korean immigrants build resilience. Resilience refers to positive adaptation to stress, encompassing coping mechanisms to overcome challenges [44]. It involves adapting to change and resisting the negative impacts of stressors. Resilient individuals display optimism, view experiences as valuable, leverage personal strengths, foster close relationships, and exhibit emotional awareness [44, 45]. Research studies have shown that high resilience correlates with improved health, accelerated productivity, and a well-developed sense of well-being [44, 45]. In addition to that, this study provided insights into the role of the church community for Korean immigrants in the United States. Participants emphasized the social support they received from the church. Beyond spiritual guidance, the church community created a space for Korean immigrants to comfortably adjust to the new environment of living in a country with many cultural differences, which, in turn, helped them gain a better sense of belonging.

### Limitations

There are a few limitations to this research study. First, purposive and snowball sampling methods might cause a selection bias, which misses embracing all different aspects of Korean immigrants’ faith and their lives. Second, the findings cannot be assumed to represent all Korean immigrants in the United States, or even the immigrant population in the Bay Area. Third, participants were very likely to portray themselves as faithful members of the religion, leading to the possibility of socially desirable responses to questions related to their Christian beliefs and their role in their lives as immigrants. Another limitation is that this study focused exclusively on religious Korean immigrants and did not include a comparison group of non-religious individuals. Therefore, we did not examine whether coping strategies or mental health outcomes differ between religious and non-religious Koreans immigrants. Since the purpose of this study was to explore the role of religion during changes in legal status rather than to identify specific mental health issues, this comparison was beyond the scope of study. In addition, these findings cannot be generalized to Korean immigrants of other faith traditions, such as Buddhists or Muslims.

### Implications for future research

Despite these limitations, the study offers several important implications. First, it highlights the critical role of Christian identity in supporting the mental health and psychological resilience of Korean immigrants navigating social and legal transitions in the United States. Churches not only provide spiritual guidance but also serve as social and cultural support networks. The findings suggest that faith-based communities may offer mental health interventions for immigrant populations. Additionally, the combination of qualitative analysis and descriptive quantitative survey findings provides invaluable insights into the Christian identity of Korean immigrants through mixed methods approaches to better understand the positive impact these beliefs have on Korean immigrants’ psychological well-being. Further research is needed to deepen our understanding of the influence of faith-based identity on the mental well-being of Asian immigrants in the United States, particularly through advanced quantitative studies that encompass both religious and non-religious participants to conduct a comparative analysis between those two groups, as well as examining how immigrants from other religions, such as Catholics, Buddhists, or Muslims, navigate faith, overcome different challenges, and show mental health outcomes in the United States. Future studies may also utilize broader sampling strategies, such as randomly recruiting through online survey panels to obtain a more representative and diverse sample of immigrants for systematic comparison.

## Conclusion

This study investigated how faith-based identity, particularly Christianity, affects the mental health and sense of belonging of Korean immigrants during their social and legal status transition in the United States. The findings highlight how faith-based identity as an important factor of spiritual health would positively influence the psychological and mental well-being of Korean immigrants. These insights contribute to the broader understanding that Christianity has offered culturally and spiritually sensitive support to Korean immigrant communities by helping them overcome challenges and establish a clear direction for their future. Future research should examine how faith-based identity influences the mental health of Korean immigrants by comparing religious and non-religious groups, exploring the experiences of those from different religions such as Buddhists or Muslims, and investigating differences in mental health during immigrants’ transition period across various regions in the United States. These factors may reveal important variations in support systems and mental health outcomes among the immigrant populations in the United States.

## Data Availability

The data that support the findings of this study are available on request from the corresponding author, CP. The data are not publicly available due to their containing information that could compromise the privacy of research participants.
